# Prevalence of Epiretinal Membrane and Macular Edema Across Uveitis Subtypes: A Retrospective Cross-Sectional Study

**DOI:** 10.3390/biomedicines14102170

**Published:** 2026-09-25

**Authors:** Chihiro Maehara, Kinya Tsubota, Masaki Asakage, Hiroshi Goto, Yoshihiko Usui

**Affiliations:** Department of Ophthalmology, Tokyo Medical University, 6-7-1 Nishi-Shinjuku, Shinjuku-ku, Tokyo 160-0023, Japan

**Keywords:** uveitis, epiretinal membrane, macular edema, optical coherence tomography, sarcoidosis, Behçet’s disease, cross-sectional study

## Abstract

**Background/Objectives:** This study aimed to determine the prevalence of epiretinal membrane (ERM) and macular edema (ME) at initial presentation across clinically defined uveitis subtypes. **Methods:** In this retrospective cross-sectional study, we reviewed clinical records from the initial visit and imaging from 3879 eyes of 2559 patients diagnosed with uveitis at Tokyo Medical University Hospital between 2011 and 2021. The prevalence of ERM and ME was estimated overall and by uveitis subtype, with additional descriptive age- and sex-stratified analyses. **Results:** ERM and ME were identified in 185/3879 eyes (4.8%) and 235/3879 eyes (6.1%), respectively. Rare subtypes with very small sample sizes showed the highest crude prevalences; however, among uveitis subtypes represented by at least 30 eyes, sarcoidosis had the highest ERM prevalence (50/363 eyes, 13.8%). By contrast, Behçet’s disease had the highest ME prevalence (44/284 eyes, 15.5%). In descriptive subgroup analyses, Behçet’s disease accounted for a greater proportion of ERM and ME in male and younger patients. By contrast, sarcoidosis accounted for a greater proportion in female and older patients. **Conclusions:** The prevalence of ERM and ME at presentation varied across uveitis subtypes; of those represented by at least 30 eyes, sarcoidosis showed the highest prevalence of ERM, whereas Behçet’s disease showed the highest prevalence of ME.

## 1. Introduction

Uveitis is a leading cause of irreversible blindness among working-age populations [1]. In the United States and other countries, the annual incidence and prevalence of uveitis range from 17.4 to 52.4 per 100,000 persons and 58.0 to 114.5 per 100,000 persons, respectively [2,3,4,5,6]. Epiretinal membrane (ERM) and macular edema (ME) are frequent macular complications and important causes of visual impairment in patients with uveitis [7,8], necessitating early diagnosis and treatment. Estimating the burden of ERM and ME is clinically relevant for screening, counseling, and resource planning. Reported rates vary across studies owing to differences in study populations, diagnostic criteria, and imaging modalities. Previous reports showed that the prevalence of ERM and ME on fluorescein angiography and optical coherence tomography (OCT) is the highest in intermediate uveitis and panuveitis [9,10]. Both ERM and ME are associated with visual impairment in patients with uveitis [8,9]. ERM and ME formation in uveitis patients is also significantly associated with anatomical types of posterior uveitis and panuveitis [11]. Reportedly, ME is a common ocular complication of intermediate uveitis [12]. Chronic uveitis is associated with higher rates of ERM and ME due to persistent inflammation. However, discrepancies in prevalence rates between studies suggest potential differences in study population, diagnostic criteria, and imaging modality. Moreover, most reports lack information on the diagnosis of specific uveitis subtypes in the patients studied; thus, differences in ERM and ME prevalence among uveitis subtypes need to be elucidated. Our literature search found only one report on ME prevalence in uveitis subtypes [8], and no report on ERM using the uveitis classification. In addition, Mustafi et al. reported that ERM was observed concurrently with or before ME development in more than 90% of eyes [11]. This suggests that ERM formation precedes ME development; its progression causes structural alterations in the inner retina.

To provide a baseline assessment during referral, we evaluated ERM and ME using imaging obtained at the initial presentation. Our objectives were to estimate the prevalence of ERM and ME at the initial visit in patients with uveitis, to compare prevalences across etiologically defined uveitis subtypes, and to describe age- and sex-related patterns. We hypothesized that prevalence and demographic distributions would vary across subtypes. Accordingly, we conducted a large-scale retrospective, single-center, cross-sectional analysis at a referral center in Japan.

## 2. Materials and Methods

### 2.1. Study Design and Setting

This paper outlines a retrospective, single-center, cross-sectional observational study based on routinely collected medical records and imaging data. We assessed ERM and ME on imaging obtained at the initial visit and estimated prevalence at the overall eye level and across etiologically defined uveitis subtypes. Analyses were performed at the eye level; age- and sex-related patterns were summarized descriptively. This study was conducted at the Department of Ophthalmology, Tokyo Medical University Hospital, a tertiary referral center in Tokyo, Japan. Consecutive patients diagnosed with uveitis between January 2011 and December 2021 were eligible. ERM and ME were assessed using imaging obtained at the initial visit. No follow-up period was defined for the assessment of ERM or ME in this cross-sectional study. Etiologic classification was performed based on the final diagnosis documented in the medical record, incorporating clinical information from subsequent visits when applicable. ERM and ME were assessed using prespecified imaging criteria applied consistently across OCT platforms, and disagreements were resolved by consensus. The study size was determined by including all eligible consecutive patients diagnosed with uveitis during the study period.

### 2.2. Subject Selection and Clinical Data Collection

This study adhered to the Declaration of Helsinki and was approved by the Tokyo Medical University Review Board (No. T2021-0001; 2 April 2021). The requirement for informed consent was waived by the Institutional Review Board, and an opt-out approach was approved. We retrospectively reviewed the electronic medical records of consecutive patients diagnosed with uveitis at our institution during the study period. Patients were excluded if they had a history of vitrectomy before the initial visit, non-uveitic pathology contributing to ERM/ME, or ungradable OCT images due to media opacity. Both eyes from the same patient were included when eligible; eligibility and outcome assessments were determined at the eye level. Clinical variables included age and sex recorded at the initial visit, the final etiologic diagnosis of uveitis documented in the medical record, and baseline ophthalmic imaging findings used to assess ERM and ME. We did not evaluate treatment status, treatment details, or inflammatory severity. To reduce selection bias, we included consecutive patients and specified eligibility criteria a priori.

### 2.3. Variables and Outcomes

The primary outcomes were the eye-level prevalence of ERM and ME at the initial visit, both overall and according to etiologically defined uveitis subtype. Secondary analyses described age- and sex-stratified patterns of ERM and ME. Age was analyzed as a continuous variable and categorically (0–24, 25–44, 45–64, and ≥65 years). The primary unit of analysis was the eye; patient-level variables, including age, sex, and uveitis subtype, were applied to each eligible eye. Prevalence was also calculated in a sensitivity analysis at the patient level to assess the potential of including both eyes from the same patients. A patient was considered affected if ERM or ME was present in at least one eye.

### 2.4. Diagnosis of Uveitis

Uveitis was classified using established diagnostic criteria and previously described clinical classification frameworks [13,14,15,16,17,18,19,20,21,22,23,24,25]. Non-infectious uveitis, including sarcoidosis, Vogt–Koyanagi–Harada disease, Behçet’s disease, Fuchs’ heterochromic iridocyclitis, tubulointerstitial nephritis and uveitis syndrome, and vitreoretinal lymphoma, was diagnosed based on established diagnostic criteria and characteristic clinical and laboratory findings [16,17,18,19,20,23]. Infectious uveitis was diagnosed based on characteristic clinical findings and identification of the causative pathogen or supportive microbiological testing, including polymerase chain reaction, serologic testing, or intraocular antibody analysis, as appropriate [26]. Acute retinal necrosis was diagnosed according to established Japanese and American Uveitis Society criteria [24,25]. Uveitis was unclassified when clinical examination and laboratory investigations did not meet the diagnostic criteria for any specific uveitis entity. Etiologic classification was based on the final diagnosis documented in the medical record, incorporating clinical and laboratory information from subsequent visits when applicable.

### 2.5. Determination of ERM and ME

All ERM and ME observations were made using data from the initial visit (baseline). The presence of ERM was primarily determined on OCT, supported by routine ophthalmic examinations, based on irregular hyperreflective signals or membranous tissue on the vitreous side of the internal limiting membrane, as described previously [9]. The ME was identified by OCT findings of cystoid intraretinal spaces in the inner nuclear layer and outer plexiform layer, with fluorescein angiography used when clinically indicated, as described in a previous report [27]. The cases in this study were evaluated and documented using either the CirrusTM HD-OCT (Carl Zeiss Meditec AG, Jena, Germany) or the RS-3000TM Advance system (NIDEK CO., LTD., Gamagori, Japan). OCT images (and fluorescein angiography, when available) were independently assessed by three investigators using prespecified criteria; discrepancies were resolved by consensus. Depending on the OCT device and available imaging protocol, both macular raster scans (5-line scans) and macular volume/map scans were reviewed for the assessment of ERM and ME.

### 2.6. Statistical Analysis

Continuous variables are summarized as mean ± standard deviation (SD); categorical variables are counts and percentages. The prevalence of ERM and ME at the initial visit was calculated on the basis of the eye overall and by etiologically defined uveitis subtypes. For the age-specific prevalence estimates shown in the result, 95% confidence intervals (CIs) were calculated using the Wilson score method. Prespecified subgroup summaries by sex and age group are presented descriptively. No formal hypothesis testing, multivariable modeling, or interaction testing was carried out because the primary aim was descriptive epidemiology. Otherwise, analyses used all available data and were performed using IBM SPSS Statistics (version 29, IBM Corp., Armonk, NY, USA).

## 3. Results

### 3.1. Prevalence of ERM and ME

During the study period, we screened consecutive patients diagnosed with uveitis at our institution. We excluded patients with prior vitrectomy before the first visit (*n* = 327), patients with non-uveitic pathology contributing to ERM/ME (*n* = 3), and patients with ungradable OCT images due to media opacity from infectious diseases (*n* = 15) (Supplemental Figure S1). The final study population comprised 2559 patients (3879 eyes) (Table 1). Supplemental Table S1 shows the prevalence of ERM at the initial visit by uveitis subtype. The prevalence of ERM at the first examination was 4.8% in all the subjects, and sympathetic ophthalmia showed the highest prevalence of ERM (30.0%, 3/10 eyes), followed by HTLV-1-associated uveitis (28.6%, 2/7 eyes) and uveitis associated with rheumatoid-related diseases (18.2%, 2/11 eyes). Because prevalence estimates based on small numbers of eyes may be unstable and appear disproportionately high, we performed an additional analysis restricted to uveitis subtypes represented by at least 30 eyes. In this analysis, sarcoidosis showed the highest ERM prevalence (13.8%, 50/363 eyes), followed by Behçet’s disease (8.5%, 24/284 eyes) and tuberculous uveitis (5.9%, 2/34 eyes) (Table 2).

Supplemental Table S2 shows the prevalence of ME at the first visit by uveitis subtype. The prevalence of ME at the first examination was 6.1% in all the subjects. The top three uveitis subtypes with the highest ME prevalence at the initial visit were sympathetic ophthalmia (30.0% (3/10 eyes)), toxocariasis (28.6% (2/7 eyes)), and uveitis associated with rheumatoid-related diseases (18.2% (2/11 eyes)). When uveitis subtypes represented by at least 30 eyes were analyzed, the highest prevalence at the first visit was 15.5% (44/284 eyes) in Behcet’s disease, followed by 10.5% (38/363 eyes) in sarcoidosis, and 7.7% (120/1561) in unclassified uveitis of unknown etiology (Table 3). In the patient-level sensitivity analysis, which defined a patient as affected when ERM or ME was present in at least one eye, ERM was present in 141 of 2559 patients (5.5%) and ME in 179 of 2559 patients (7.0%), compared with the corresponding eye-level prevalences of 4.8% and 6.1%, respectively. These patient-level estimates were similar to the eye-level estimates and did not materially alter the main findings.

### 3.2. Sex- and Age-Stratified Analyses

In prespecified sex-stratified analyses, female patients showed a higher prevalence of both ERM and ME than male patients (Figure 1). Detailed results on specific subtypes by sex are provided in Supplementary Tables S3–S6. Briefly, among eyes with ERM or ME, Behçet’s disease accounted for a larger proportion of cases in male patients. By contrast, sarcoidosis accounted for a larger proportion in female patients, after excluding unclassified uveitis. These findings suggest that the distribution of uveitis-associated macular complications may differ by sex, although the analyses were descriptive and limited by small sample sizes in several subtypes.

ERM prevalence increased with age and was highest in patients aged ≥65 years (9.6%, 95% CI: 7.9–11.7%). The prevalence of ME was higher in the two older age groups, reaching 7.5% in patients aged 45–64 years and 7.7% in those aged ≥65 years (Figure 2). Detailed age-specific subtype distributions are shown in Supplementary Tables S7 and S8. After excluding unclassified uveitis, Behçet’s disease was the most frequent diagnosis associated with ERM or ME in younger age groups, while sarcoidosis was more prominent in older age groups. Because several disease categories included only a small sample of eyes, the age-stratified findings should be considered exploratory and interpreted with caution.

## 4. Discussion

This large retrospective, single-center, cross-sectional study of consecutive uveitis patients at the initial visit was designed to estimate the prevalence of epiretinal membrane (ERM) and macular edema (ME). It also aimed to compare these prevalences across etiologically defined uveitis subtypes, describing age- and sex-related patterns. Overall, ERM and ME were present in 185 of 3879 eyes (4.8%) and 235 of 3879 eyes (6.1%), respectively. In the patient-level sensitivity analysis, defining a patient as affected when ERM or ME was present in at least one eye, ERM was present in 141 of 2559 patients (5.5%) and ME in 179 of 2559 patients (7.0%). These patient-level estimates were similar to the corresponding eye-level estimates. Among common uveitis subtypes represented by at least 30 eyes, sarcoidosis showed the highest ERM prevalence, whereas Behçet’s disease showed the highest ME prevalence, highlighting substantial heterogeneity by etiology. In stratified analyses, Behçet’s disease predominated in male and younger patients, while sarcoidosis was more common in female and older patients. Prior reports indicate higher prevalence of ERM and ME ranging from 18 to 51.6% and 19.1 to 25%, respectively [9,10,11,28]. This is because the data in this study were derived from the initial visits, and the prevalence of ME and ERM is likely to increase as follow-up progresses. ERM and ME were extremely rare findings in patients with Posner–Schlossman syndrome, uveitis with Epstein–Barr virus, endogenous fungal endophthalmitis, HIV retinitis, progressive outer retinal necrosis, acute zonal occult outer retinopathy, drug-induced uveitis, lens-induced uveitis, geographic choroiditis, multiple evanescent white dot syndrome, and uveitis with granulomatosis and polyangiitis. The absence of ERM or ME in certain diseases may relate to factors such as inflammation confined to the anterior segment of the eye, a short interval between disease onset and the initial examination, and the small number of cases in the present cohort.

Studies suggest that ERM associated with uveitis may histologically differ from idiopathic ERM [29], and the prevalence of ERM in uveitic eyes has been reported to be higher than that observed in population-based studies of idiopathic ERM [9,30]. However, the reported prevalence of idiopathic ERM in the literature ranges widely from 1.02 to 28.9%. This variability may relate to differences in country of study, race, and age [31]. Therefore, age should be considered when interpreting ERM prevalence in patients with uveitis, because it significantly increases with age in the general population [32]. Previous OCT-based studies have shown that ERM is more frequent in intermediate, posterior, and panuveitis than in anterior uveitis [9,10]. In the present study, some types of infectious uveitis and uveitis with chronic inflammation had the highest ERM prevalence. The age-stratified analysis also showed that ERM tended to increase with age.

The exact mechanism of ERM formation is not fully understood [33]. A study suggests a significant association between ERM formation and the expression levels of specific cytokines or growth factors in the intraocular fluids [34]. The underlying mechanism is speculated to be changes in intraocular proteins resulting from the production of inflammatory cytokines associated with uveitis, which contribute to ERM formation [35,36]. We postulate that ERM formation in intraocular inflammation is caused by increases in specific cytokines and growth factors in certain uveitis subtypes such as sarcoidosis [37]. Indeed, intraocular diseases can be diagnosed based on the immune mediator profile in the AH [38]; thus, changes in intraocular levels of immune mediators, including cytokines associated with intraocular inflammation, may be important in monitoring ERM formation.

In uveitis, ME often develops as the blood–retinal barrier breaks down, and ME is a common cause of eyesight loss, accounting for 41% of visual impairment and 29% of blindness in uveitis [39,40]. The prevalence of diabetic ME in patients with diabetes has been reported to range from 3.8 to 15.9% [41,42,43], leading to the speculation that the development of ME due to uveitis may vary significantly depending on the type of uveitis. According to a previous report, ME is more frequently observed in patients with older onset of uveitis [44]. Conversely, ME is less likely to develop in pediatric cases compared to adult cases of uveitis [44]. This is consistent with our age-stratified analysis, in which ME prevalence was higher in patients aged 45–64 years and ≥65 years than in the two younger age groups. Compared with acute uveitis, chronic uveitis is more frequently associated with ME, similarly to ERM [44]. Inflammatory cytokines such as vascular endothelial growth factor (VEGF) and tumor necrosis factor (TNF)α have been suggested to disrupt the integrity of the inner blood–retinal barrier formed by capillary endothelial cells; damage to tight junctions of endothelial cells results in accumulation of extracellular fluid [45,46].

The top three uveitis subtypes with the highest prevalence of ERM at the initial examination were sympathetic ophthalmia, HTLV-1-associated uveitis, and uveitis related to rheumatoid diseases. The top three for ME were sympathetic ophthalmia, toxocariasis, and uveitis related to rheumatoid diseases. Sympathetic ophthalmia and uveitis related to rheumatoid diseases are common in both lists, and both are non-infectious diseases that may require long-term treatment. Sympathetic ophthalmia manifests as panuveitis, and a large retrospective case series reported ME in around 4–9% of patients at baseline and 9–12% at 12 months, depending on disease duration [47]. The risk of recurrence and complications associated with the chronic course are considered problematic for patients with sympathetic ophthalmia. Although reports specifically describing ERM in sympathetic ophthalmia are limited, the high prevalence of ERM observed in this study may result from the chronic course of sympathetic ophthalmia. However, because sympathetic ophthalmia is rare and only a small number of cases have been reported in the Japanese population [48], the present result remains to be verified. When analyzing diseases with a sufficient number of cases (30 or more), sarcoidosis and Behcet’s disease were among the top three highest prevalences for both ERM and ME. Thus, our study demonstrates that sarcoidosis has a higher prevalence of ERM and ME, consistent with previous reports [49,50].

Major limitations of our study should be acknowledged. First, the retrospective, single-center design may have introduced selection and referral bias. Because all patients were recruited from a tertiary uveitis referral center, diagnostically challenging or more severe cases may have been overrepresented. In addition, a relatively large proportion of patients had unclassified uveitis (40.1%), which limits the completeness of etiologic characterization and should be considered when interpreting the subtype-specific findings. Second, because several uveitis subtypes included only a small number of eyes, their prevalence estimates may be imprecise and should be interpreted with caution. Third, this study did not systematically evaluate treatment status, inflammatory severity, duration of inflammation, or prior ocular surgery, although these factors may influence the development of ERM and ME [9]. In addition, the present study evaluated only the presence or absence of ERM and did not assess its morphological severity. Therefore, we could not determine the extent to which treatment or disease activity influenced the presence of ERM and ME at the initial visit. Future longitudinal studies incorporating standardized ERM grading with disease activity, duration of inflammation, treatment history, and surgical history are warranted. Overall, the present findings should be interpreted as prevalence estimates at the time of referral rather than cumulative risks over the disease course. Because we examined multiple uveitis entities and performed descriptive subgroup analyses by sex and age, some observed differences, particularly for rare subtypes, may reflect imprecision or chance variation. These subtype-specific patterns should therefore be regarded as exploratory and warrant confirmation in multicenter cohorts with longitudinal follow-up and standardized assessment of disease activity and treatment.

## 5. Conclusions

In this retrospective cross-sectional study of patients with uveitis, ERM and ME were present in 4.8% and 6.1% of eyes at the initial visit, respectively. Among uveitis subtypes represented by at least 30 eyes, sarcoidosis showed the highest ERM prevalence (13.8%), whereas Behçet’s disease showed the highest ME prevalence (15.5%). Age-stratified analyses showed that ERM prevalence increased with age and was highest in patients aged ≥65 years, whereas ME prevalence was higher in the two older age groups. In addition, Behçet’s disease accounted for a greater proportion of ERM and ME in younger and male patients, whereas sarcoidosis accounted for a greater proportion in older and female patients. These findings suggest that the prevalence and etiologic distribution of ERM and ME differ by uveitis subtype, age, and sex.

## Figures and Tables

**Figure 1:**
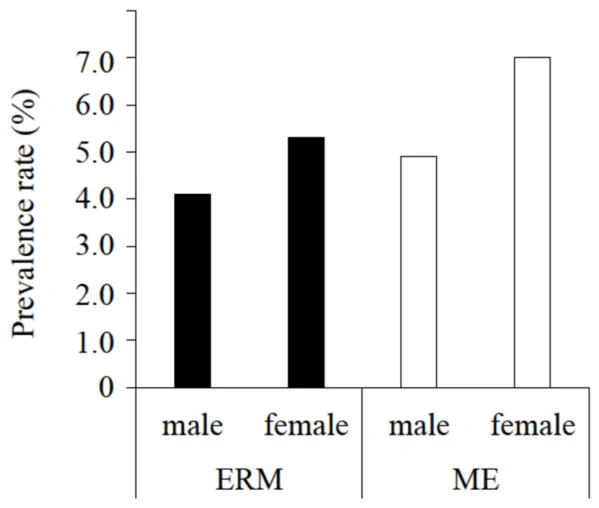
Comparison of epiretinal membrane (ERM) and macular edema (ME) prevalence in male and female patients with uveitis. Female patients with uveitis show a higher prevalence of both ERM and ME.

**Figure 2:**
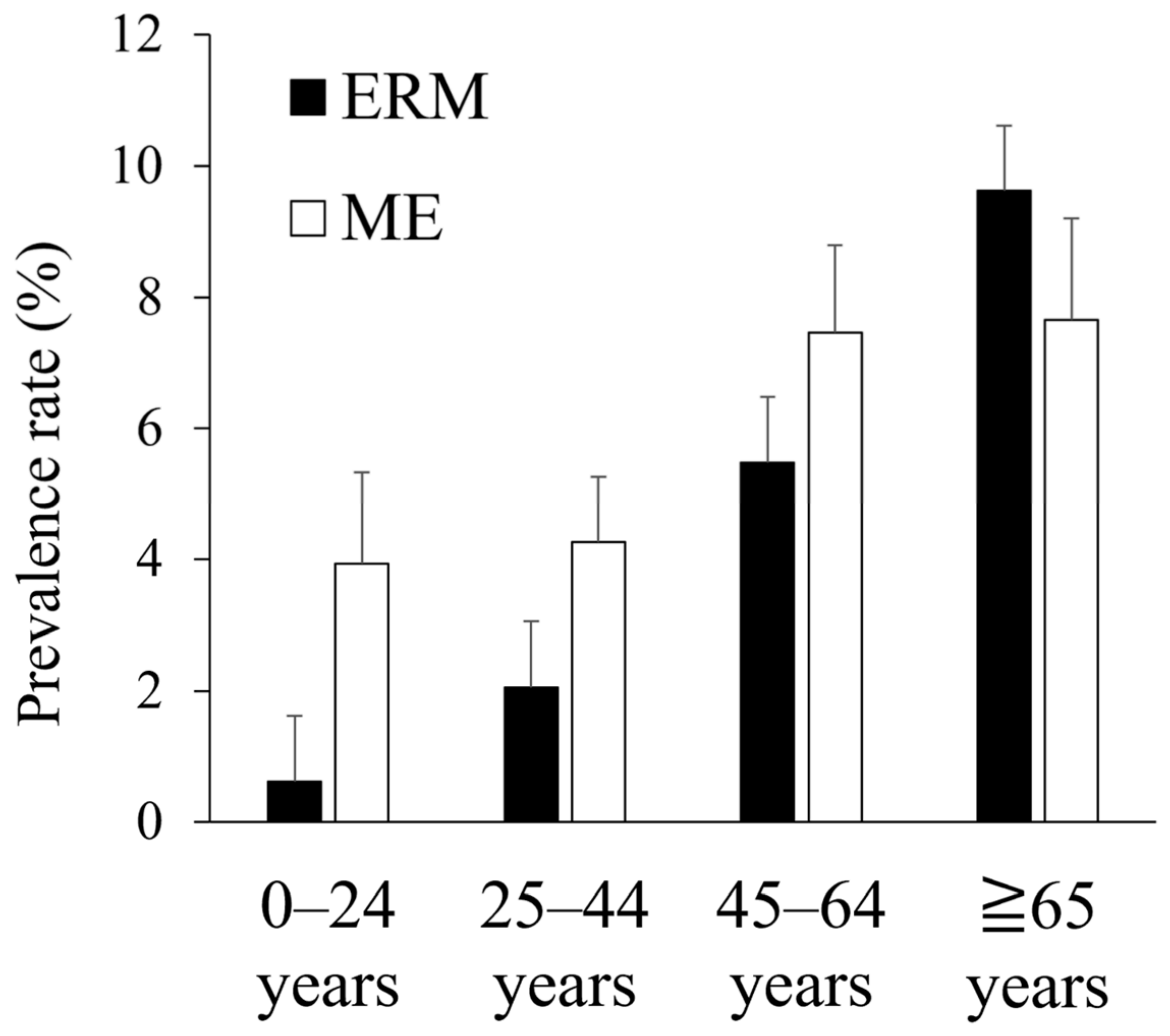
Age-specific prevalence of epiretinal membrane (ERM) and macular edema (ME) in patients with uveitis. Error bars represent 95% confidence intervals calculated using the Wilson score method. ERM prevalence increased with age, whereas ME prevalence was higher in the two older age groups.

**Table 1:** Patient demographics.

Characteristics	No. or Mean ± SD
Number of patients (eyes), *n*	2559 (3879)
Sex: male/female, *n*	1174/1385
Age, years	48.4 ± 19.3
Number of eyes in each uveitis subtype	
Unclassified uveitis, *n*	1561
Vogt–Koyanagi–Harada disease, *n*	483
Sarcoidosis, *n*	363
Behcet disease, *n*	284
Acute anterior uveitis, *n*	191
Herpetic uveitis, *n*	182
Vitreoretinal lymphoma, *n*	131
Acute retinal necrosis, *n*	65
Toxoplasmosis, *n*	54
Syphilis, *n*	51
Posner–Schlossman syndrome, *n*	50
Fuchs’ heterochromic iridocyclitis, *n*	43
CMV retinitis, n	43
Iridocyclitis in young girls, *n*	40
Diabetic uveitis, *n*	39
Tuberculous uveitis, *n*	34
The tubulointerstitial nephritis and uveitis syndrome, *n*	33
Uveitis in bowel disease, *n*	24
Multiple evanescent white dot syndrome, *n*	22
Geographic choroiditis, *n*	20
Multifocal posterior pigment epitheliopathy, *n*	19
Uveitis in juvenile idiopathic arthritis, *n*	17
Punctate inner choroidopathy, *n*	12
Multifocal choroiditis with panuveitis, *n*	12
Uveitis associated with rheumatoid-related diseases, *n*	11
Endogenous fungal endophthalmitis, *n*	11
Sympathetic ophthalmia, *n*	10
Sclerouveitis, *n*	9
Endogenous bacterial endophthalmitis, *n*	9
Acute posterior multifocal placoid pigment epitheliopathy, *n*	9
Uveitis in psoriatic arthritis, *n*	8
Lens-induced uveitis, *n*	8
Toxocariasis, *n*	7
HTLV-1-associated uveitis, *n*	7
Drug-induced uveitis, *n*	5
HIV retinitis, *n*	4
Acute zonal occult outer retinopathy, *n*	4
Progressive outer retinal necrosis, *n*	2
Uveitis with granulomatosis with polyangiitis, *n*	1
Uveitis with Epstein–Barr virus, *n*	1

**Table 2:** Prevalence of epiretinal membrane in patients with uveitis subtypes represented by at least 30 eyes.

Uveitis Subtype	No. of Patients	No. of Patients with ERM	Patient-Level Prevalence	No. of Eyes	No. of Eyes with ERM	Eye-Level Prevalence	Age, Years Mean ± SD	Male, *n* (%)	Female, *n* (%)
Sarcoidosis	191	31	16.2%	363	50	13.80%	55.1 ± 17.6	68 (35.6%)	123 (64.4%)
Behcet disease	161	19	11.8%	284	24	8.50%	38.9 ± 14.6	92 (57.1%)	69 (42.9%)
Tuberculous uveitis	22	1	4.5%	34	2	5.90%	49.5 ± 18.9	11 (50.0%)	11 (50.0%)
Diabetic uveitis	30	2	6.7%	39	2	5.10%	51.2 ± 11.9	21 (70.0%)	9 (30.0%)
Acute retinal necrosis	61	3	4.9%	65	3	4.60%	54.9 ± 19.6	25 (41.0%)	36 (59.0%)
Syphilis	34	2	5.9%	51	2	3.90%	41.5 ± 11.9	29 (85.3%)	5 (14.7%)
Fuchs’ heterochronic iridocyclitis	40	1	2.5%	43	1	2.30%	42.0 ± 12.2	18 (45.0%)	22 (55.0%)
CMV retinitis	31	1	3.2%	43	1	2.30%	54.6 ± 20.2	26 (83.9%)	5 (16.1%)
Toxoplasmosis	48	1	2.1%	54	1	1.90%	43.5 ± 19.6	25 (52.1%)	23 (47.9%)
Vitreoretinal lymphoma	80	2	2.5%	131	2	1.50%	67.9 ± 11.7	37 (46.2%)	43 (53.8%)
Vogt–Koyanagi–Harada disease	247	4	1.6%	483	4	0.80%	46.1 ± 14.7	116 (47.0%)	131 (53.0%)
Acute anterior uveitis	164	0	0.0%	191	0	0%	41.1 ± 12.9	88 (53.7%)	76 (46.3%)
Herpetic uveitis	175	0	0.0%	182	0	0%	58.7 ± 17.8	96 (54.9%)	79 (45.1%)
Posner–Schlossman syndrome	47	0	0.0%	50	0	0%	59.6 ± 14.2	29 (61.7%)	18 (38.3%)
Iridocyclitis in young girls	21	0	0.0%	40	0	0%	13.3 ± 4.7	0 (0.0%)	21 (100.0%)
Tubulointerstitial nephritis and uveitis syndrome	17	0	0.0%	33	0	0%	14.4 ± 3.5	6 (35.3%)	11 (64.7%)
Unclassified uveitis	1025	67	6.5%	1561	83	5.30%	48.6 ± 20.0	407 (39.7%)	618 (60.3%)

ERM: epiretinal membrane; CMV: cytomegalovirus.

**Table 3:** Prevalence of macular edema in patients with uveitis subtypes represented by at least 30 eyes.

Uveitis Subtype	No. of Patients	No. of Patients with ME	Patient-Level Prevalence	No. of Eyes	No. of Eyes with ME	Eye-Level Prevalence	Age, Years Mean ± SD	Male, *n* (%)	Female, *n* (%)
Behcet disease	161	28	17.4%	284	44	15.50%	38.9 ± 14.6	92 (57.1%)	69 (42.9%)
Sarcoidosis	191	29	15.2%	363	38	10.50%	55.1 ± 17.6	68 (35.6%)	123 (64.4%)
Diabetic uveitis	30	2	6.7%	39	2	5.10%	51.2 ± 11.9	21 (70.0%)	9 (30.0%)
Toxoplasmosis	48	2	4.2%	54	2	3.70%	43.5 ± 19.6	25 (52.1%)	23 (47.9%)
The tubulointerstitial nephritis and uveitis syndrome	17	1	5.9%	33	1	3.00%	14.4 ± 3.5	6 (35.3%)	11 (64.7%)
Iridocyclitis in young girls	21	1	4.8%	40	1	2.50%	13.3 ± 4.7	0 (0.0%)	21 (100.0%)
Vitreoretinal lymphoma	80	2	2.5%	131	3	2.30%	67.9 ± 11.7	37 (46.2%)	43 (53.8%)
Syphilis	34	1	2.9%	51	1	2.00%	41.5 ± 11.9	29 (85.3%)	5 (14.7%)
Acute retinal necrosis	61	1	1.6%	65	1	1.50%	54.9 ± 19.6	25 (41.0%)	36 (59.0%)
Acute anterior uveitis	164	2	1.2%	191	2	1%	41.1 ± 12.9	88 (53.7%)	76 (46.3%)
Herpetic uveitis	175	1	0.6%	182	1	0.50%	58.7 ± 17.8	96 (54.9%)	79 (45.1%)
Vogt–Koyanagi–Harada disease	247	1	0.4%	483	1	0.20%	46.1 ± 14.7	116 (47.0%)	131 (53.0%)
Posner–Schlossman syndrome	47	0	0.0%	50	0	0%	59.6 ± 14.2	29 (61.7%)	18 (38.3%)
Fuchs’ heterochronic iridocyclitis	40	0	0.0%	43	0	0%	42.0 ± 12.2	18 (45.0%)	22 (55.0%)
CMV retinitis	31	0	0.0%	43	0	0%	54.6 ± 20.2	26 (83.9%)	5 (16.1%)
Tuberculous uveitis	22	0	0.0%	34	0	0%	49.5 ± 18.9	11 (50.0%)	11 (50.0%)
Unclassified uveitis	1025	93	9.1%	1561	120	7.70%	48.6 ± 20.0	407 (39.7%)	618 (60.3%)
ME = macular edema;									
CMV = cytomegalovirus.									

## Data Availability

The data presented in this study are available on request from the corresponding author due to privacy.

## Supplementary Materials

The following supporting information can be downloaded at: https://www.mdpi.com/article/10.3390/biomedicines14102170/s1. Figure S1: Study flow diagram.; Table S1: Prevalence of epiretinal membrane in patients with various uveitis subtypes; Table S2: Prevalence of macular edema in patients with various uveitis subtypes; Table S3: Prevalence of epiretinal membrane in patients with various uveitis subtypes: males only; Table S4: Prevalence of epiretinal membrane in patients with various uveitis subtypes: females only; Table S5: Prevalence of macular edema in patients with various uveitis subtypes: males only; Table S6: Prevalence of macular edema in patients with various uveitis subtypes: females only; Table S7: Age-stratified analysis of the prevalence of epiretinal membrane in patients with various uveitis subtypes; Table S8: Age-stratified analysis of the prevalence of macular edema in patients with various uveitis subtypes.
