# Supplementary material for: Prevalence of Epiretinal Membrane and Macular Edema Across Uveitis Subtypes: A Retrospective Cross-Sectional Study
Source: Biomedicines. 2026 Sep 25;14(10):2170. doi: 10.3390/biomedicines14102170 (PMC13701911; doi:10.3390/biomedicines14102170)
Supplement: Supplementary file 1 [file biomedicines-14-02170-s001.zip › Supplemental Table S1.pdf]

Supplemental Table S1. Prevalence of epiretinal membrane in patients with various uveitis subtypes

Uveitis subtype	No. of patients	No. of eyes (a)	No. of eyes with ERM (b)	b/a (%)
Sympathetic ophthalmia	8	10	3	(30%)
HTLV-1-associated uveitis	4	7	2	(28.6%)
Uveitis associated with rheumatoid-related diseases	6	11	2	(18.2%)
Toxocariasis	6	7	1	(14.3%)
Sarcoidosis	191	363	50	(13.8%)
Uveitis in psoriatic arthritis	6	8	1	(12.5%)
Behcet disease	161	284	24	(8.5%)
Punctate inner choroidopathy	7	12	1	(8.3%)
Tuberculous uveitis	22	34	2	(5.9%)
Diabetic uveitis	30	39	2	(5.1%)
Acute retinal necrosis	61	65	3	(4.6%)
Syphilis	34	51	2	(3.9%)
Fuchs' heterochromic iridocyclitis	40	43	1	(2.3%)
CMV retinitis	31	43	1	(2.3%)
Toxoplasmosis	48	54	1	(1.9%)
Vitreoretinal lymphoma	80	131	2	(1.5%)
Vogt-Koyanagi-Harada disease	247	483	4	(0.8%)
Acute anterior uveitis	164	191	0	(0%)
Herpetic uveitis	175	182	0	(0%)
Posner-Schlossman syndrome	47	50	0	(0%)
Iridocyclitis in young girls	21	40	0	(0%)
The tubulointerstitial nephritis and uveitis syndrome	17	33	0	(0%)
Uveitis in bowel disease	17	24	0	(0%)
Multiple evanescent white dot syndrome	22	22	0	(0%)
Geographic choroiditis	13	20	0	(0%)
Multifocal posterior pigment epitheliopathy	12	19	0	(0%)
Uveitis in juvenile idiopathic arthritis	11	17	0	(0%)
Multifocal choroiditis with Panuveitis	7	12	0	(0%)
Endogenous fungal endophthalmitis	6	11	0	(0%)
Acute posterior multifocal placoid pigment epitheliopathy	6	9	0	(0%)
Sclerouveitis	7	9	0	(0%)
Endogenous bacterial endophthalmitis	6	9	0	(0%)
Lens-induced uveitis	8	8	0	(0%)
Drug-induced uveitis	3	5	0	(0%)

HIV retinitis	2	4	0	(0%)
Acute zonal occult outer retinopathy	4	4	0	(0%)
Progressive outer retinal necrosis	2	2	0	(0%)
Uveitis with Epstein-Barr virus	1	1	0	(0%)
Uveitis with granulomatosis with polyangiitis	1	1	0	(0%)
Unclassified uveitis	1,025	1,561	83	(5.3%)
Total	2,559	3,879	185	(4.8%)

ERM= epiretinal membrane

HTLV-1= human T-lymphotropic virus type-1

CMV= cytomegalo virus

HIV= human immunodeficiency virus
