# Supplementary material for: Prevalence of Epiretinal Membrane and Macular Edema Across Uveitis Subtypes: A Retrospective Cross-Sectional Study
Source: Biomedicines. 2026 Sep 25;14(10):2170. doi: 10.3390/biomedicines14102170 (PMC13701911; doi:10.3390/biomedicines14102170)
Supplement: Supplementary file 1 [file biomedicines-14-02170-s001.zip › Supplemental Table S2.pdf]

Supplemental Table S2. Prevalence of macular edema in patients with various uveitis subtypes

Uveitis subtype	No. of patients	No. of eyes (a)	No. of eyes with ME (b)	b/a (%)
Sympathetic Ophthalmia	8	10	3	(30%)
Toxocariasis	6	7	2	(28.6%)
Uveitis associated with rheumatoid-related diseases	6	11	2	(18.2%)
Punctate inner choroidopathy	7	12	2	(16.7%)
Behcet disease	161	284	44	(15.5%)
HTLV-1-associated uveitis	4	7	1	(14.3%)
Uveitis in psoriatic arthritis	6	8	1	(12.5%)
Sclerouveitis	7	9	1	(11.1%)
Acute posterior multifocal placoid pigment epitheliopathy	6	9	1	(11.1%)
Sarcoidosis	191	363	38	(10.5%)
Uveitis in bowel disease	17	24	2	(8.3%)
Multifocal choroiditis with panuveitis	7	12	1	(8.3%)
Uveitis in juvenile idiopathic arthritis	11	17	1	(5.9%)
Multifocal posterior pigment epitheliopathy	12	19	1	(5.3%)
Diabetic uveitis	30	39	2	(5.1%)
Toxoplasmosis	48	54	2	(3.7%)
The tubulointerstitial nephritis and uveitis syndrome	17	33	1	(3.0%)
Iridocyclitis in young girls	21	40	1	(2.5%)
Vitreoretinal lymphoma	80	131	3	(2.3%)
Syphilis	34	51	1	(2.0%)
Acute retinal necrosis	61	65	1	(1.5%)
Acute anterior uveitis	164	191	2	(1%)
Herpetic uveitis	175	182	1	(0.5%)
Vogt-Koyanagi-Harada disease	247	483	1	(0.2%)
Posner-Schlossman syndrome	47	50	0	(0%)
Fuchs' heterochronic iridocyclitis	40	43	0	(0%)
CMV retinitis	31	43	0	(0%)
Tuberculous uveitis	22	34	0	(0%)
Multiple evanescent white dot syndrome	22	22	0	(0%)
Geographic choroiditis	13	20	0	(0%)
Endogenous fungal endophthalmitis	6	11	0	(0%)
Endogenous bacterial endophthalmitis	6	9	0	(0%)
Lens-induced uveitis	8	8	0	(0%)
Drug-induced uveitis	3	5	0	(0%)

Acute zonal occult outer retinopathy	4	4	0	(0%)
HIV retinitis	2	4	0	(0%)
Progressive outer retinal necrosis	2	2	0	(0%)
Uveitis with Epstein-Barr virus	1	1	0	(0%)
Uveitis with granulomatosis with polyangiitis	1	1	0	(0%)
Unclassified uveitis	1,025	1,561	120	(7.7%)
Total	2,559	3,879	235	(6.1%)

ME= macular edema

HTLV-1=human T-lymphotropic virus type-1

CMV= cytomegalo virus

HIV= human immunodeficiency virus
