# Supplementary material for: Prevalence of Epiretinal Membrane and Macular Edema Across Uveitis Subtypes: A Retrospective Cross-Sectional Study
Source: Biomedicines. 2026 Sep 25;14(10):2170. doi: 10.3390/biomedicines14102170 (PMC13701911; doi:10.3390/biomedicines14102170)
Supplement: Supplementary file 1 [file biomedicines-14-02170-s001.zip › Supplemental Table S3.pdf]

Supplemental Table S3. Prevalence of epiretinal membrane in patients with various uveitis subtypes: males only

Uveitis subtype	No. of eyes (a)	No. of eyes with ERM (b)	b/a (%)	b/total eyes with ERM (%)
HTLV-1-associated Uveitis	3	2	66.7	2.9
Sympathetic Ophthalmia	5	3	60.0	4.3
Behcet disease	92	19	20.7	27.5
Punctate inner choroidopathy	4	1	25.0	1.4
Uveitis in psoriatic arthritis	5	1	20.0	1.4
Tuberculous uveitis	19	2	10.5	2.9
Diabetic uveitis	26	2	7.7	2.9
Sarcoidosis	126	9	7.1	13.0
CMV retinitis	37	1	2.7	1.4
Syphilis	44	1	2.3	1.4
Vogt-Koyanagi-Harada disease	226	2	0.9	2.9
Unclassified uveitis	584	26	4.5	37.7

ERM= epiretinal membrane

HTLV-1= human T-lymphotropic virus type-1

CMV= cytomegalovirus
