# Supplementary material for: Prevalence of Epiretinal Membrane and Macular Edema Across Uveitis Subtypes: A Retrospective Cross-Sectional Study
Source: Biomedicines. 2026 Sep 25;14(10):2170. doi: 10.3390/biomedicines14102170 (PMC13701911; doi:10.3390/biomedicines14102170)
Supplement: Supplementary file 1 [file biomedicines-14-02170-s001.zip › Supplemental Table S4.pdf]

Supplemental Table S4. Prevalence of epiretinal membrane in patients with various uveitis subtypes: females only

Uveitis subtype	No. of eyes (a)	No. of eyes with ERM (b)	b/a (%)	b/total eyes with ERM (%)
Uveitis associated with rheumatoid-related diseases	5	2	40.0	1.7
Toxocariasis	4	1	25.0	0.9
Sarcoidosis	237	41	17.3	35.3
Syphilis	7	1	14.3	0.9
Acute retinal necrosis	38	3	7.9	2.6
Fuchs' heterochronic iridocyclitis	25	1	4.0	0.9
Toxoplasmosis	26	1	3.8	0.9
Vitreoretinal lymphoma	73	2	2.7	1.7
Behcet disease	192	5	2.6	4.3
Vogt-Koyanagi-Harada disease	257	2	0.8	1.7
Unclassified uveitis	977	57	5.8	49.1

ERM= epiretinal membrane
