# Supplementary material for: Prevalence of Epiretinal Membrane and Macular Edema Across Uveitis Subtypes: A Retrospective Cross-Sectional Study
Source: Biomedicines. 2026 Sep 25;14(10):2170. doi: 10.3390/biomedicines14102170 (PMC13701911; doi:10.3390/biomedicines14102170)
Supplement: Supplementary file 1 [file biomedicines-14-02170-s001.zip › Supplemental Table S5.pdf]

Supplemental Table S5. Prevalence of macular edema in patients with various uveitis subtypes: males only

Uveitis subtype	No. of eyes (a)	No. of eyes with ME (b)	b/a (%)	b/ total eyes with ME (%)
Sympathetic ophthalmia	5	2	40.0	2.4
Toxocariasis	3	1	33.3	1.2
HTLV-1-associated Uveitis	3	1	33.3	1.2
Punctate inner choroidopathy	4	1	25.0	1.2
Behcet disease	92	23	25.0	27.4
Uveitis in psoriatic arthritis	5	1	20.0	1.2
Uveitis in bowel disease	16	2	12.5	2.4
Multifocal posterior pigment epitheliopathy	12	1	8.3	1.2
Diabetic uveitis	26	2	7.7	2.4
Sarcoidosis	126	7	5.6	8.3
Vitreoretinal lymphoma	58	3	5.2	3.6
Toxoplasmosis	28	1	3.6	1.2
Syphilis	44	1	2.3	1.2
Acute anterior uveitis	88	1	1.1	1.2
Vogt-Koyanagi-Harada disease	226	1	0.4	1.2
Unclassified uveitis	584	36	6.2	42.9

ME= macular edema

HTLV-1= human T-lymphotropic virus type-1
