# Supplementary material for: Prevalence of Epiretinal Membrane and Macular Edema Across Uveitis Subtypes: A Retrospective Cross-Sectional Study
Source: Biomedicines. 2026 Sep 25;14(10):2170. doi: 10.3390/biomedicines14102170 (PMC13701911; doi:10.3390/biomedicines14102170)
Supplement: Supplementary file 1 [file biomedicines-14-02170-s001.zip › Supplemental Table S6.pdf]

Supplemental Table S6. Prevalence of macular edema in patients with various uveitis subtypes: females only

Uveitis subtype	No. of eyes (a)	No. of eyes with ME (b)	b/a (%)	b/total eyes with ME (%)
Uveitis associated with rheumatoid-related diseases	5	2	40.0	1.4
Acute posterior multifocal placoid pigment epitheliopathy	4	1	25.0	0.7
Toxocariasis	4	1	25.0	0.7
Sympathetic ophthalmia	5	1	20.0	0.7
Sarcoidosis	237	31	13.1	20.9
Sclerouveitis	7	1	14.3	0.7
Syphilis	7	1	14.3	0.7
Punctate inner choroidopathy	8	1	12.5	0.7
Multifocal Choroiditis with Panuveitis	10	1	10.0	0.7
Behcet disease	192	17	8.9	11.5
Uveitis in juvenile idiopathic arthritis	15	1	6.7	0.7
The tubulointerstitial nephritis and uveitis syndrome	22	1	4.5	0.7
Toxoplasmosis	26	1	3.8	0.7
Acute retinal necrosis	38	1	2.6	0.7
Iridocyclitis in young girls	40	1	2.5	0.7
Herpetic uveitis	79	1	1.3	0.7
Acute anterior uveitis	103	1	1.0	0.7
Unclassified uveitis	977	84	8.6	56.8
