# Supplementary material for: Prevalence of Epiretinal Membrane and Macular Edema Across Uveitis Subtypes: A Retrospective Cross-Sectional Study
Source: Biomedicines. 2026 Sep 25;14(10):2170. doi: 10.3390/biomedicines14102170 (PMC13701911; doi:10.3390/biomedicines14102170)
Supplement: Supplementary file 1 [file biomedicines-14-02170-s001.zip › Supplemental Table S7.pdf]

Supplemental Table S7. Age-stratified analysis of the prevalence of epiretinal membrane in patients with various uveitis subtypes

Uveitis subtype	No. of eyes (a)	No. of eyes with ERM (b)	b/a (%)	b/total eyes with ERM in each age group (%)
0-24 years				
Uveitis in psoriatic arthritis	3	1	33.3	33.3
Punctate inner choroidopathy	5	1	20.0	33.3
Unclassified uveitis	237	1	0.4	33.3
25-44 years				
Toxocariasis	5	1	20.0	3.7
Syphilis	28	2	7.1	7.4
Tuberculous uveitis	28	2	7.1	7.4
CMV retinitis	16	1	6.3	3.7
Behcet disease	148	8	5.4	29.6
Acute retinal necrosis	19	1	5.3	3.7
Fuchs' heterochromic iridocyclitis	27	1	3.7	3.7
Sarcoidosis	97	1	1.0	3.7
Vogt-Koyanagi-Harada disease	208	1	0.5	3.7
Unclassified uveitis	408	9	2.2	33.3
45-64 years				
HTLV-1-associated Uveitis	6	2	33.3	2.9
Sympathetic ophthalmia	4	1	25.0	1.5
Tuberculous uveitis	10	2	20.0	2.9
Behcet disease	73	11	15.1	16.2
Sarcoidosis	128	19	14.8	27.9
Acute retinal necrosis	19	2	10.5	2.9
Diabetic uveitis	19	2	10.5	2.9
Toxoplasmosis	18	1	5.6	1.5
Unclassified uveitis	504	28	5.6	41.2
≥ 65 years				
Uveitis associated with rheumatoid-related diseases	5	2	40.0	2.3
Behcet disease	19	5	26.3	5.7
Sarcoidosis	124	30	24.2	34.1
Sympathetic ophthalmia	6	1	16.7	1.1

Vogt-Koyanagi-Harada disease	59	3	5.1	3.4
Vitreoretinal lymphoma	83	2	2.4	2.3
Unclassified uveitis	412	45	10.9	51.1

ERM= epiretinal membrane

CMV= cytomegalovirus

HTLV-1= human T-lymphotropic virus type-1
