# Supplementary material for: Prevalence of Epiretinal Membrane and Macular Edema Across Uveitis Subtypes: A Retrospective Cross-Sectional Study
Source: Biomedicines. 2026 Sep 25;14(10):2170. doi: 10.3390/biomedicines14102170 (PMC13701911; doi:10.3390/biomedicines14102170)
Supplement: Supplementary file 1 [file biomedicines-14-02170-s001.zip › Supplemental Table S8.pdf]

Supplemental Table S8. Age-stratified analysis of the prevalence of macular edema in patients with various uveitis subtypes

Uveitis subtype	No. of eyes (a)	No. of eyes with ME (b)	b/a (%)	b/total eyes with ME in each age group (%)
0-24 years				
Uveitis in psoriatic arthritis	3	1	33.3	5.3
Punctate inner choroidopathy	5	1	20.0	5.3
Behcet disease	44	5	11.4	26.3
Uveitis in juvenile idiopathic arthritis	17	1	5.9	5.3
The tubulointerstitial nephritis and uveitis syndrome	33	1	3.0	5.3
Iridocyclitis in young girls	40	1	2.5	5.3
Unclassified uveitis	237	9	3.8	47.4
25-44 years				
Acute posterior multifocal placoid pigment epitheliopathy	2	1	50.0	1.9
Punctate inner choroidopathy	5	1	20.0	1.9
Toxocariasis	5	1	20.0	1.9
Behcet disease	148	29	19.6	55.8
Diabetic uveitis	13	1	7.7	1.9
Syphilis	28	1	3.6	1.9
Sarcoidosis	97	3	3.1	5.8
Unclassified uveitis	408	15	3.7	28.8
45-64 years				
Toxocariasis	2	1	50.0	1.1
Sympathetic ophthalmia	4	1	25.0	1.1
HTLV-1-associated Uveitis	6	1	16.7	1.1
Uveitis in bowel disease	13	2	15.4	2.2
Multifocal Choroiditis with Panuveitis	7	1	14.3	1.1
Sarcoidosis	128	18	14.1	20.2
Toxoplasmosis	18	2	11.1	2.2
Multifocal posterior pigment epitheliopathy	11	1	9.1	1.1
Behcet disease	73	6	8.2	6.7
Acute retinal necrosis	19	1	5.3	1.1
Diabetic uveitis	19	1	5.3	1.1
Vitreoretinal lymphoma	44	2	4.5	2.2
Acute anterior uveitis	59	1	1.7	1.1
Vogt-Koyanagi-Harada disease	191	1	0.5	1.1

Unclassified uveitis	504	50	9.9	56.2
≥ 65 years				
Uveitis associated with rheumatoid-related diseases	5	2	40.0	2.7
Behcet disease	19	5	26.3	6.8
Sclerouveitis	5	1	20.0	1.4
Sympathetic ophthalmia	6	1	16.7	1.4
Sarcoidosis	124	17	13.7	23.0
Herpetic uveitis	83	1	1.2	1.4
Vitreoretinal lymphoma	83	1	1.2	1.4
Unclassified uveitis	412	46	11.2	62.2

ME= macular edema

HTLV-1= human T-lymphotropic virus type-1
